# Time Series Resolution of the Fish Necrobiome Reveals a Decomposer Succession Involving Toxigenic Bacterial Pathogens

**DOI:** 10.1128/mSystems.00145-20

**Published:** 2020-04-28

**Authors:** Briallen Lobb, Rhiannon Hodgson, Michael D. J. Lynch, Michael J. Mansfield, Jiujun Cheng, Trevor C. Charles, Josh D. Neufeld, Paul M. Craig, Andrew C. Doxey

**Affiliations:** a Department of Biology, University of Waterloo, Waterloo, Ontario, Canada; b Metagenom Bio Life Science Inc., Waterloo, Ontario, Canada; MIT

**Keywords:** necrobiome, microbiome, decomposition, wastewater, rainbow darter, *Aeromonas*, aerolysin, community profiling, fish, metagenomics, thanatomicrobiome, toxins

## Abstract

Despite progress understanding microbial communities involved in terrestrial vertebrate decomposition, little is known about the microbial decomposition of aquatic vertebrates from a functional and environmental context. Here, we analyzed temporal changes in the “necrobiome” of rainbow darters, which are common North American fish that are sensitive indicators of water quality. By combining 16S rRNA gene and shotgun metagenomic sequence data from four time points, we studied the progression of decomposers from both taxonomic and functional perspectives. The 16S rRNA gene profiles revealed strong community succession, with early decomposition stages associated with *Aeromonas* and *Clostridium* taxa and later stages dominated by members of the *Rikenellaceae* (i.e., *Alistipes*/*Acetobacteroides* genera). These results were reproducible and independent of environmental perturbation, given that exposure to wastewater treatment plant effluent did not substantially influence the necrobiome composition of fish or the associated water sample microbiota. Metagenomic analysis revealed significant changes throughout decomposition in degradation pathways for amino acids, carbohydrates/glycans, and other compounds, in addition to putrefaction pathways for production of putrescine, cadaverine, and indole. Binning of contigs confirmed a predominance of *Aeromonas* genome assemblies, including those from novel strains related to the pathogen Aeromonas veronii. These bins of *Aeromonas* genes also encoded known hemolysin toxins (e.g., aerolysin) that were particularly abundant early in the process, potentially contributing to host cell lysis during decomposition. Overall, our results demonstrate that wild-caught fish have a reproducible decomposer succession and that the fish necrobiome serves as a potential source of putative pathogens and toxigenic bacteria.

**IMPORTANCE** The microbial decomposition of animal tissues is an important ecological process that impacts nutrient cycling in natural environments. We studied the microbial decomposition of a common North American fish (rainbow darters) over four time points, combining 16S rRNA gene and shotgun metagenomic sequence data to obtain both taxonomic and functional perspectives. Our data revealed a strong community succession that was reproduced across different fish and environments. Decomposition time point was the main driver of community composition and functional potential; fish environmental origin (upstream or downstream of a wastewater treatment plant) had a secondary effect. We also identified strains related to the putative pathogen Aeromonas veronii as dominant members of the decomposition community. These bacteria peaked early in decomposition and coincided with the metagenomic abundance of hemolytic toxin genes. Our work reveals a strong decomposer succession in wild-caught fish, providing functional and taxonomic insights into the vertebrate necrobiome.

## Abstract (precis)

The microbial decomposition of animal tissues is an important ecological process that impacts nutrient cycling in natural environments. We studied the microbial decomposition of a common North American fish (rainbow darters) over four time points, combining 16S rRNA gene and shotgun metagenomic sequence data to obtain both taxonomic and functional perspectives. Our data revealed a strong community succession that was reproduced across different fish and environments. Decomposition time point was the main driver of community composition and functional potential; fish environmental origin (upstream or downstream of a wastewater treatment plant) had a secondary effect. We also identified strains related to the putative pathogen Aeromonas veronii as dominant members of the decomposition community. These bacteria peaked early in decomposition and coincided with the metagenomic abundance of hemolytic toxin genes. Our work reveals a strong decomposer succession in wild-caught fish, providing functional and taxonomic insights into the vertebrate necrobiome.

## INTRODUCTION

The decomposition of animal tissues is a fundamental ecological process that impacts nutrient cycling and species composition in terrestrial and aquatic ecosystems. Vertebrate tissue decomposition creates a unique ecological niche supporting a wide variety of specialized decomposer species, including insects, predators, and microorganisms. These species form an interconnected community whose combined activities lead to the decomposition of an organism from its initial death to the complete degradation of its exterior and internal contents.

The microbial communities involved in decomposition, including bacteria derived from the surrounding environment (e.g., water, soil) and the host (e.g., digestive tract and lungs), are collectively referred to as the “necrobiome” (from nekrós, the Greek word for dead body) (1), or alternatively, the “thanatomicrobiome” (from Thanatos, the Greek god of death) (2). Studies of necrobiome structure and function in several model systems (e.g., human, cow, pig, and mouse) have revealed strong microbial succession with distinct taxonomic and functional shifts linked to the phases of tissue decomposition (3–8). After cellular autolysis breaks down tissue following death, anaerobic bacteria such as *Clostridium* spp. increase in relative abundance and metabolize available carbohydrates and proteins from the body, producing organic acids and gas (9). Functional shifts occur; these shifts include increases in catabolic pathways, carbohydrate and energy metabolism, nitrogen cycling, and processes related to bacterial invasion. Foul-smelling compounds associated with the process of putrefaction are also produced as by-products of fermentation and amino acid decomposition, including putrescine, cadaverine, and indole. Because putatively pathogenic bacteria proliferate within vertebrate necrobiomes, such as Clostridium botulinum (10), it has been proposed that bacterial toxins secreted by these bacteria may play roles in decomposition by interfering with host cellular functions (11).

Although much knowledge of necrobiome community structure and function has come from studies of terrestrial mammals, less is known about the structure, function, and dynamics of decomposition in aquatic ecosystems. Previous studies of fish carcass decomposition demonstrate that as in terrestrial systems, both macroinvertebrates and microorganisms play important roles as aquatic decomposers (12, 13). Microbial analysis of forage fish (i.e., menhaden) carrion decomposing for 48 h in an estuary-like environment revealed a necrobiome dominated by members of the *Firmicutes*, *Bacteroidetes*, and *Gammaproteobacteria* (14). Similarly, a freshwater study of salmon carcasses reported that members of the *Proteobacteria*, *Firmicutes*, and *Bacteroidetes* dominated carcass decomposition (15). Despite these previous studies characterizing the taxonomic shifts associated with fish decomposition, much more work is needed to explore the microbial communities and their functions within fish necrobiomes and their associated aquatic ecosystems. Particularly important research questions include the following. (i) What is the composition of aquatic vertebrate necrobiomes and how does it change over time? (ii) How do changes in environmental parameters affect necrobiome communities? (iii) What metabolic activities/functions are present in necrobiome communities and how do they change over time?

In this study, to gain insights into these questions, we focused on the rainbow darter (Etheostoma caeruleum) as a model organism to profile microbial decomposer community succession from both taxonomic and functional perspectives. Rainbow darters are a common North American fish found in streams and small- to medium-sized river, and they have high site fidelity and sensitivity to changes in water quality (16–18). Thus, they represent ideal targets for exploring the role of host-associated and location-specific microbial communities present before and after death. Rainbow darters are affected physiologically by disturbances of the river ecosystem, such as wastewater treatment plant (WWTP) effluent inputs and urbanization (16–18). These anthropogenic factors affect rivers by introducing pollutants and altering the balance of available nutrients (18–20), thus influencing fish physiology and their associated microbiomes. Using 16S rRNA sequencing combined with metagenomics, we study both the taxonomic and functional succession of the rainbow darter necrobiome community. We also compare necrobiomes between two different locations in the Grand River (southwestern Ontario, Canada), upstream and downstream of a WWTP, allowing us to analyze community members and their functional potential both spatially and temporally. Studying necrobiome-associated microbial communities provides a unique way to better understand links to aquatic health, fish physiology, and ecosystem dynamics.

## RESULTS AND DISCUSSION

### Time series community profiling of fish necrobiomes.

**FIG 1:**
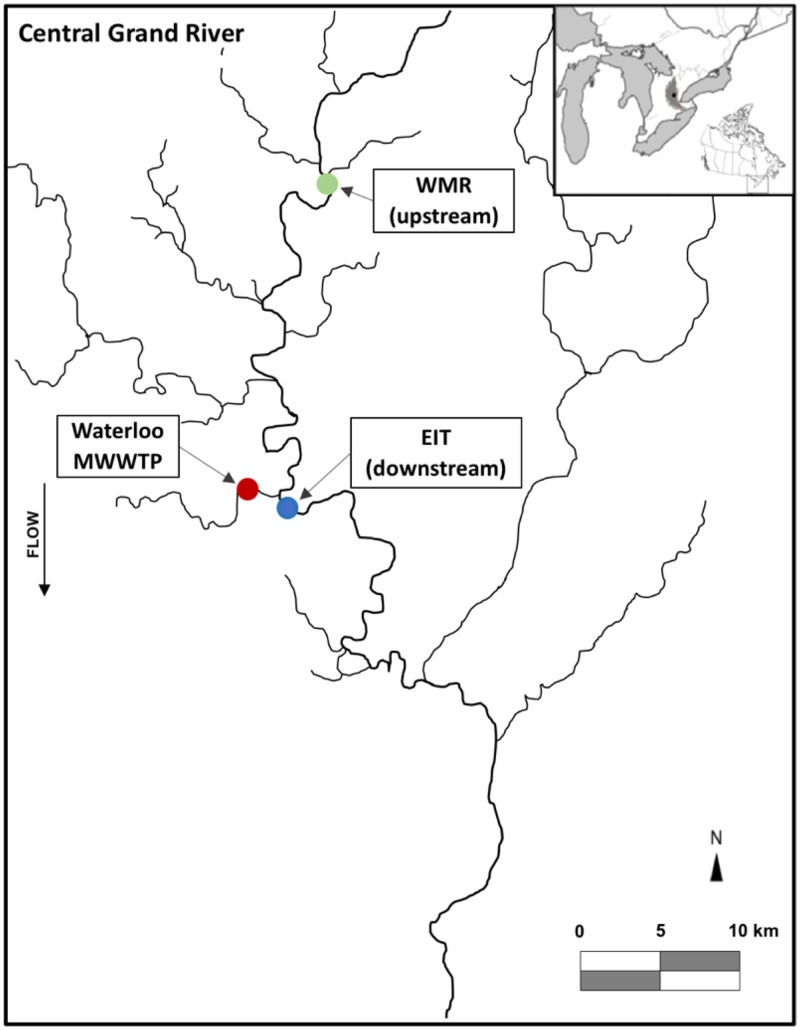
Map showing sampling locations of Grand River fish for metagenomic analysis. The municipal wastewater treatment plant (WWTP) for the city of Waterloo, Canada, and the two sampling locations, upstream at West Montrose (WMR) and downstream at the Economic Insurance Trail (EIT), are displayed.

**FIG 2:**
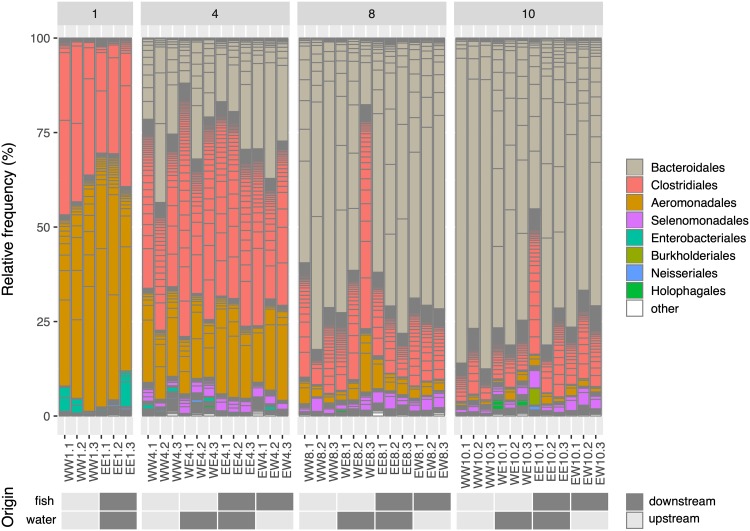
Relative frequency of ASVs within each sample colored by taxonomic order. Samples are sorted by decomposition time (1 day, 4 days, 8 days, and 10 days). The fish and water/sediment origin of the samples are displayed at the bottom of the figure, with upstream referring to the WMR site and downstream referring to the EIT site. Low-relative-abundance taxonomic orders are grouped into “other.”

**FIG 3:**
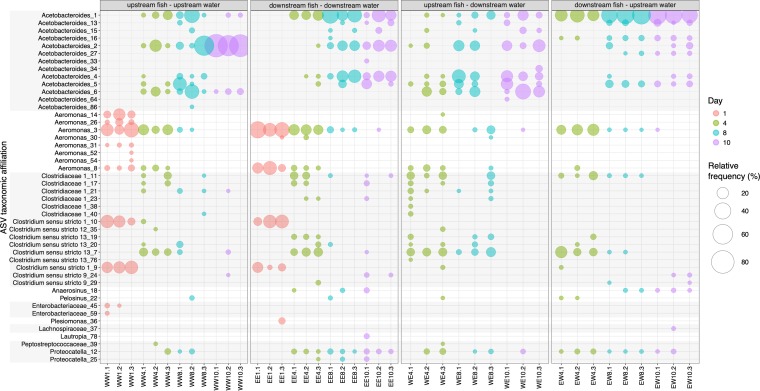
Bubble-plot depicting the relative abundance (as a percentage) of ASVs in the fish necrobiome at four time points. Light gray boxes indicate shared family level taxonomic affiliation. Bubbles are displayed only if the ASV taxonomic affiliation was ≥2%. For other ASVs, see Data Set S1A in the supplemental material. Bubbles are colored by decomposition time (days).

To examine the structure and temporal succession of aquatic vertebrate necrobiomes, we performed a 16S rRNA-based study of decomposing fish at different time points and locations. We collected female rainbow darters (Etheostoma caeruleum) from the Grand River in Waterloo, Ontario, Canada, both upstream and downstream of the Waterloo wastewater treatment plant (WWTP) (Fig. 1). Individual fish were subjected to decomposition with river water and sediment at room temperature for 1, 4, 8, and 10 days in sterile containers that acted as microcosms of a natural decomposition environment. Sample 16S rRNA gene profiles for fish decomposition microbiomes (“necrobiomes”) for these four time points and two water/sediment sources revealed reproducible microbial communities among independent replicates and also between environments (i.e., fish and water source; Fig. 2 and 3). This microbial succession was apparent at the order level of taxonomy (Fig. 2) and at the level of amplicon sequence variants (ASVs) (Fig. 3), although variation in ASV composition was evident among fish samples and environments (Fig. 3).

10.1128/mSystems.00145-20.10 DATA SET S1 (A) ASV table; (B) differential relative abundances; (C) Bin_4 VFanalyzer results; (D) Bin_11 VFanalyzer results. Download Data Set S1, XLSX file, 0.4 MB. Copyright © 2020 Lobb et al. 2020 Lobb et al. This content is distributed under the terms of the Creative Commons Attribution 4.0 International license.

Although infectious lesions began to form on fish sampled on day 1, “bloat-stage” decomposition associated with anaerobic microbial decomposition was not visually apparent until later time points (see Fig. S1b in the supplemental material). The day 1 decomposer communities were composed predominantly of taxa affiliated with *Aeromonas* and *Clostridium*, and to a lesser extent by members of the *Enterobacteriales*. All of these bacterial groups are commonly associated with fish gut microbial communities (21–24), especially *Aeromonas* within freshwater fish microbiota (25, 26). Species of *Clostridium sensu stricto* 1 have proteolytic activities and thus may be associated with a more carnivorous diet (24), consistent with the predominantly insectivorous diets of rainbow darters (27). The taxa that were abundant in day 1 fish (e.g., *Clostridium sensu stricto* 1 sp. ASV 9 and 10, and *Aeromonas* sp. ASV 3 and 8) decreased in relative abundance over the course of decomposition from 11 to 28% on day 1 to < 2% on day 10.

10.1128/mSystems.00145-20.1 FIG S1 Decomposition setup and images of fish decay. (a) Example of the mason jars used for decay of female rainbow darter (*Etheostoma caeruleum*) in a microcosm of the Grand River. (b) Representative images of decay of female rainbow darter for enrichment of the necrobiome at 1, 4, 8, and 10 days. Download FIG S1, TIF file, 2.2 MB. Copyright © 2020 Lobb et al. 2020 Lobb et al. This content is distributed under the terms of the Creative Commons Attribution 4.0 International license.

Fish sampled on day 4 were associated with anaerobic bloat-stage decomposition and advanced tissue degradation (Fig. S1b). Consistent with previous studies of decomposition using other fish species (14, 15), dominant day 4 bacterial phyla detected were *Proteobacteria*, *Firmicutes*, and *Bacteroidetes* (Fig. 2). Compared to day 1 samples, there were considerable changes in degrader community composition for day 4 profiles, with substantial increases in *Acetobacteroides* ASVs (family *Rikenellaceae*, order *Bacteroidales*) from < 2% on day 1 up to 21% for some ASVs (Fig. 2 and 3). The *Acetobacteroides* genus was associated with 45 distinct ASVs across all samples, with 13 ASVs at a relative abundance of ≥2%. Characterized *Acetobacteroides* members are fermentative, mesophilic, strictly anaerobic, and capable of metabolizing carbohydrates and producing acetate, H_2_, and CO_2_ as end products (28). These bacteria also classify under the “Blvii28 wastewater-sludge group” according to the SILVA database. Following day 4, taxa affiliated with *Acetobacteroides* were the dominant decomposer group, increasing to a relative abundance of as much as 87% in the decomposer community by the final day 10 sampling. Distinct *Acetobacteroides* ASVs dominated the day 10 decomposer community for fish collected from each of the two river locations (Fig. 3). *Acetobacteroides* ASV 2 increased to a relative abundance 77% in the fish and water pairing collected upstream of the Waterloo WWTP, whereas *Acetobacteroides* ASV 1 increased to a relative abundance of 51% in fish collected downstream of the WWTP and left to decompose in water/sediment collected from upstream. These results indicate an influence of the sampled environment on species- or strain-level variations in decomposer communities. The day 4 decomposer communities were also associated with an increase in taxa affiliated with the *Selenomonadales* order, including *Pelosinus* and *Anaerosinus* genera (Fig. 3), which persisted throughout the decomposition process but at low relative abundance. The relative abundance of *Selenomonadales* increased from 0.0037% on day 1 to 3.8% on day 4, 3.5% on day 8, and 3.3% on day 10.

### Influence of spatial location on fish necrobiome succession.

**FIG 4:**
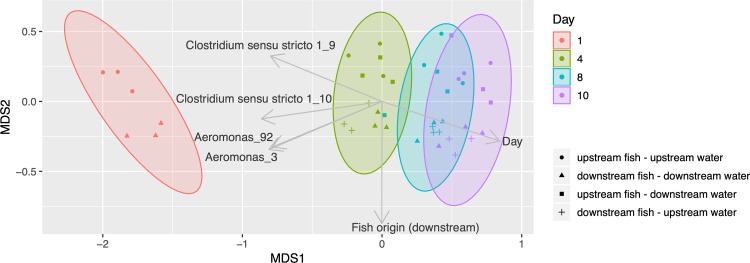
A nonmetric multidimensional scaling (NMDS) ordination of necrobiomes based on microbial community composition, using Bray-Curtis distances generated from ASV frequency profiles. Stress is 0.098. Together, 99% of the variance is represented based on the *R*^2^ value between distance in ordination space and distance in the original matrix. Vectors with *R*^2^ values greater than 0.7 were shown on the plot. Ellipses are colored by decomposition time (days).

Based on sample ordination, necrobiome 16S rRNA gene profiles separated primarily by time point, with distinct microbial communities associated with different stages of tissue decomposition (Fig. 4). Necrobiomes also separated according to sample type, demonstrating distinct profiles for fish that originated upstream versus downstream of the WWTP, but this separation was less apparent than separation based on time. There was no strong effect of water/sediment origin on sample separation. This pattern was found for samples in which fish decomposed in water from the same location and in “swap” experiments in which fish were transferred into sediment/water samples derived from different original locations (e.g., upstream WWTP fish decomposing in downstream WWTP water). Even when isolating the effect of decomposition time, no effect of water/sediment sample origin was detected (Fig. S2). Thus, necrobiomes appear to be influenced primarily by factors occurring prior to decomposition, such as the living fish microbiomes, physiological states, or other fish-environment interactions.

10.1128/mSystems.00145-20.2 FIG S2 NMDS ordination of necrobiomes after 4, 8, and 10 days of decomposition based on microbial community composition with Bray-Curtis distances. The ordinations in the top and bottom row for each time point are the same. The ellipses for the top row were calculated for sample fish origin, while the ellipses for the bottom row were calculated for sample water/sediment origin. A strong agreement between the ordination space and the distance matrix was observed for time points 4 days, 8 days, and 10 days (*R*^2^ = 0.984, 0.989, and 0.991, respectively) and the stress values are 0.127, 0.103, and 0.097, respectively. Download FIG S2, TIF file, 1.0 MB. Copyright © 2020 Lobb et al. 2020 Lobb et al. This content is distributed under the terms of the Creative Commons Attribution 4.0 International license.

**FIG 5:**
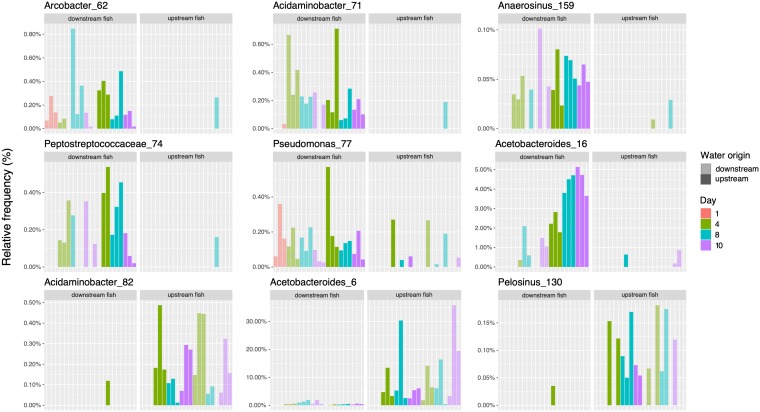
Differentially abundant ASVs between necrobiomes from upstream versus downstream WWTP fish samples. Differential relative abundance was calculated based on pairwise rank sum tests of relative frequency (community proportion). The top ASVs with adjusted *P* values of less than 0.05 are shown. Bars are colored by decomposition time (days), with opacity determined by water origin.

In order to further investigate the influence of fish origin location on decomposer microbial community differences, we calculated differentially abundant taxa (*P* < 0.05, Mann-Whitney U test) among fish necrobiomes associated with upstream and downstream WWTP fish sources (Fig. 5; for additional ASVs, see Table S2 in the supplemental material; for additional statistical methods, see Data Set S1B). Necrobiome-associated taxa with significantly increased relative abundances (*P* < 0.05 and fold changes of >3) in fish collected upstream of the WWTP include species of *Acetobacteroides* (ASV 6) and *Pelosinus* (ASV 130) (Fig. 5). Taxa with significantly increased relative abundances in downstream fish samples include *Anaerosinus* (ASV 159), *Peptostreptococcaceae* (ASV 74), *Arcobacter* (ASV 62), and *Pseudomonas* (ASV 77). However, these ASVs were low-abundance organisms with a relative abundance of less than 1%. Characterized *Peptostreptococcaceae* species are anaerobic bacteria that include pathogens associated with tissue infections and antibiotic resistance (29). Known *Arcobacter* species are aerotolerant and include human and animal pathogens that have been found in groundwater and water reservoirs (30–32). An additional taxonomic group that increased in relative abundance in necrobiomes originating from fish collected from downstream of the WWTP was *Tolumonas* (e.g., ASV 176), a genus with member species associated with the production of toluene from phenol precursors, which are known wastewater pollutants (33, 34). Further investigation into the contribution of wastewater effluent on fish decomposition would be helpful to confirm that these differences were indeed linked to WWTP effluent, especially because these putative pathogenic bacteria may contribute to a portion of the energetic costs of fish living downstream of WWTPs (18).

In samples with both fish and water/sediment originating downstream of the WWTP, we also observed higher relative abundance of ASVs associated with *Plesiomonas* and *Lautropia* genera (Fig. 3). Again, these ASVs had low (<1%) relative abundance. Known *Plesiomonas* species associate with aquatic habitats, cause human infections associated with uncooked shellfish, and have been implicated in infectious outbreaks in regions, including Canada (35, 36). *Lautropia* species have been isolated from the oral cavities of immunocompromised individuals suffering from HIV and cystic fibrosis (37, 38).

### Metagenomic binning and analysis of decomposition pathways.

To explore the genomes and genome-encoded metabolic/functional potential of the necrobiomes, we performed metagenomic sequencing on one replicate for each condition (14 total). Subsequent assembly and binning resulted in four MAGs (metagenome-assembled genomes) with >85% completion and <5% redundancy. We examined the taxonomic composition of the MAGs using MetAnnotate (39). These MAGs included two genomes affiliated with *Alistipes* (*Rikenellaceae*), a genome annotated as Aeromonas veronii, and a *Selenomonadaceae*-associated genome (Table 1). The bins are consistent with ASVs identified by 16S rRNA gene sequencing, corresponding to *Acetobacteroides* (*Rikenellaceae*), *Aeromonas*, and various members of the *Selenomonadales* (Fig. 2 and 3). Other ASVs identified by 16S rRNA gene sequencing were also recovered in the lower-quality MAGs (Table 1). One bin was affiliated with the genus *Pseudomonas*, and another bin was affiliated with the family *Rikenellaceae*.

**TABLE 1:** Bins obtained from metagenomic sequencing of fish necrobiomes

Bin name	Completion (%)	Redundancy (%)	GC content (%)	Total length (Mb)	Gene count	Contig count	Taxonomic affiliation (predicted)
Bin_4	98.6	0.7	60.7	3.85	3,855	784	*Bacteria*, *Proteobacteria*, *Gammaproteobacteria*, *Aeromonadales*, *Aeromonadaceae*, *Aeromonas*, Aeromonas veronii
Bin_9	97.1	1.4	47.5	2.25	2,216	402	*Bacteria*, *Firmicutes*, *Negativicutes*, *Selenomonadales*, *Selenomonadaceae*, *Propionispira*
Bin_3	87.1	2.2	47.0	2.64	2,467	801	*Bacteria*, *Bacteroidetes*, *Bacteroidia*, *Bacteroidales*, *Rikenellaceae*, *Alistipes*
Bin_10	92.8	2.2	44.0	3.26	2,882	368	*Bacteria*, *Bacteroidetes*, *Bacteroidia*, *Bacteroidales*, *Rikenellaceae*, *Alistipes*
Bin_7	38.8	7.9	61.4	0.78	1,187	628	*Bacteria*, *Proteobacteria*, *Gammaproteobacteria*, *Pseudomonadales*, *Pseudomonadaceae*, *Pseudomonas*
Bin_2	25.2	1.4	48.2	1.71	1,872	960	*Bacteria*, *Bacteroidetes*, *Bacteroidia*, *Bacteroidales*, *Rikenellaceae*

The relative abundance of Bin_4 (Aeromonas veronii) decreased throughout decomposition from an average relative abundance of 3.7 (day 1) to an average relative abundance of 0.14 (day 10), consistent with our 16S rRNA data (Fig. S3a). Because *Aeromonas* has been associated with fish gut microbiomes (40–44), it is possible that Bin_4 and other *Aeromonas* taxa were initially derived from the fish guts and were important only for early stage decomposition. In contrast, Bin_3 (*Rikenellaceae* family) may represent a late-stage decomposer because its relative abundance increased in metagenomes from days 8 to 10 of decomposition (average relative abundance of 3.9 on day 8 to an average relative abundance 5.1 on day 10; Fig. S3a). In the downstream fish-upstream sediment/water set, both *Rikenellaceae*-affiliated bins (Bin_3 and Bin_10) were similar in relative abundance, implying site-specific influences on the relative abundance of different *Rikenellaceae*-affiliated taxa, consistent with 16S rRNA gene data for *Acetobacteroides* ASVs (Fig. 3). Phylogenetic analysis of the two *Rikenellaceae*-associated bins revealed that Bin_3 was more closely related to Acetobacteroides hydrogenigenes RL-C and Bin_10 was more closely related to *Alistipes* sp. strain ZOR0009 (Fig. S3b). Bin_9 (*Propionispira*) was present at low (0.0 to 0.54 average on days 1 to 10; Fig. S3a) relative abundance, close to the sample’s mean coverage across the entire course of decomposition, consistent with the abundance patterns seen for *Selenomonadales* based on 16S rRNA gene data (Fig. 2).

10.1128/mSystems.00145-20.3 FIG S3 Metagenomic bin relative abundance and phylogenetic analysis of Bin_3 and Bin_10. (a) Relative abundance of four high-quality binned genomes across each necrobiome sample. Relative abundance was computed as mean bin coverage/mean sample coverage. Mean coverage was calculated per base pair using Anvi’o (66). (b) RAxML tree using the LG likelihood model made from concatenated single-copy core protein sequences detected with Anvi’o (Campbell et al. set [70]). The tree outgrouped with *Lentimicrobium saccharophilum*. Acetobacteroides hydrogenigenes, representatives of *Alistipes* strains, and all uncharacterized *Alistipes* isolates were used for this tree and sourced from NCBI Genome. This tree was visualized with iTOL (69). Download FIG S3, TIF file, 0.6 MB. Copyright © 2020 Lobb et al. 2020 Lobb et al. This content is distributed under the terms of the Creative Commons Attribution 4.0 International license.

**FIG 6:**
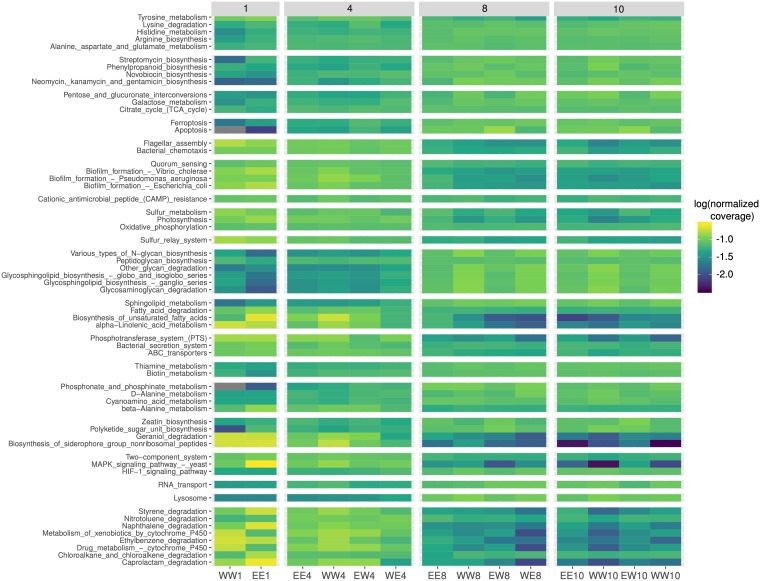
Selected KEGG pathways displaying significant differential relative abundance across the course of decomposition. Pathways were selected that had an unadjusted *P* value of <0.03 after a Kruskall-Wallis test comparing decomposition time (1, 4, 8 , and 10 days). Shown is the log_10_ value of the fractional coverage of the pathway with respect to the total coverage across all the pathways in the sample. Total pathway coverage is also proportionally normalized across every sample. Note that some pathways are based on a few representative genes. For example, coverage of the photosynthesis pathway is mainly derived from genes encoding sodium ion pumps.

Using a KEGG analysis of assembled contigs and binned metagenomes, we examined metabolic pathway potentials associated with decomposition samples. The resulting functional profiles had a highly similar grouping in ordination space compared to the 16S rRNA gene community profiles, whereby samples grouped primarily based on decomposition time point (Fig. S4). Analysis of specific KEGG pathways revealed patterns consistent with a functional succession, mirroring the taxonomic succession described earlier (Fig. 6). Pollutant degradation pathways for polyaromatic hydrocarbons such as naphthalene, styrene, and nitrotoluene showed increased relative abundances on day 1 (13% on average) compared to subsequent time points (6.2% on average). The initial fish bacterial community may have been enriched for microorganisms that could degrade river water contaminants, which can originate from both anthropogenic and natural sources and bioaccumulate in fish (45–47). Naphthalene degradation in polluted sediment-water systems can be accomplished through several bacterial pathways, and bioremediation of this toxic molecule by native organisms is currently being studied (48–50). Various biofilm formation pathways were also proportionally abundant (13%) within day 1 metagenomes (Fig. 6), possibly reflecting skin and gut community functions originating prior to decomposition. Degrading river water contaminants and skin and gut biofilm formation may be functions that are more important for the bacterial communities living with their fish host and dealing with possibly contaminated river water than for the necrobiome that formed in our closed system after the fish’s death.

10.1128/mSystems.00145-20.4 FIG S4 NMDS ordination of metagenomic functional profiles with Bray-Curtis distances calculated based on KEGG pathway frequencies. A strong agreement between the ordination space and the distance matrix was observed (*R*^2^ = 0.996), and the stress value is 0.063. Download FIG S4, TIF file, 0.5 MB. Copyright © 2020 Lobb et al. 2020 Lobb et al. This content is distributed under the terms of the Creative Commons Attribution 4.0 International license.

Glycan metabolism generally increased in coverage from early stages (2.4% on day 1) to later stages of decomposition (10%). Glycan degradation pathways (e.g., glycosaminoglycans) increased in coverage by days 8 and 10, which may be involved in decomposition of fish skin and intestinal mucins. Late-stage increases in streptomycin, phenylpropanoid, novobiocin, neomycin, kanamycin, and gentamicin biosynthesis pathways (2.4-fold change from day 1 to 10) were also detected, implying that the remaining microorganisms by day 10 possess increased potential for antibiotic synthesis.

These metagenome-wide functional patterns closely matched the functional potentials of individual *Aeromonas* (early stage) and *Rikenellaceae* (late stage) bins, when taking into consideration their shifts in relative abundance through the time course (Fig. S5). Genes belonging to pollutant degradation pathways were present in the *Aeromonas* bin yet mostly absent from other MAGs with lower relative abundance from days 1 and 4 metagenomes. Likewise, biofilm formation pathway genes had a 6.2-fold-higher frequency in the *Aeromonas* bin compared to the *Acetobacteroides*/*Alistipes* bins. In contrast, antibiotic biosynthesis pathway genes had a 2.5-fold-higher frequency in the *Rikenellaceae*-associated bins, in addition to multiple key glycan degradation genes. Thus, the detected shifts in functional profiles were in part due to the hand-off microbial community dominance from *Aeromonas* to *Rikenellaceae*. It is important to note that these apparent late-stage functional shifts could also be important for earlier phases when *Rikenellaceae* initially began to increase in relative abundance.

10.1128/mSystems.00145-20.5 FIG S5 Count of KEGG annotations mapping to the corresponding KEGG pathways in Fig. 6 across each MAG. Shown is the log_10_ value of the fractional frequency of the pathway with respect to the total across all the pathways in the sample. The total pathway coverage is also proportionally normalized across every sample. Download FIG S5, TIF file, 1.0 MB. Copyright © 2020 Lobb et al. 2020 Lobb et al. This content is distributed under the terms of the Creative Commons Attribution 4.0 International license.

Our data suggest strong *Acetobacteroides* dominance in late-stage rainbow darter necrobiomes (Fig. 2 and 3). Because related species have been implicated in anaerobic sugar fermentation (28), we investigated the two MAGs affiliated with these bacteria for glycolytic enzymes. Both Bin_3 and Bin_10 possess a complete glycolysis pathway as well as l-lactate dehydrogenase for anaerobic fermentation (Fig. S6). Bin 3 genes also encode pyruvate dehydrogenase, aldehyde dehydrogenase, and enzymes for conversion of d-fructose, d-fructose-1-phosphate (d-fructose-1P), and d-mannose-6P to glycolysis precursors. Based on a previous analysis of decomposition pathways (51), Bin_3 and Bin_10 genes also encode components of potential pathways for production of indole (EC 4.1.99.1), putrescine (EC 3.5.3.11), and spermidine (EC 2.5.1.6 and 2.5.1.16), in addition to histidine degradation (EC 4.3.1.3, 4.2.1.49, and 3.5.3.8, Bin_10 only).

10.1128/mSystems.00145-20.6 FIG S6 End of the KEGG glycolysis/gluconeogenesis pathway for *Rikenellaceae* Bin_10 and Bin_3. Green indicates the presence of a match to that enzyme. Images were generated using KEGG (67). Download FIG S6, TIF file, 1.4 MB. Copyright © 2020 Lobb et al. 2020 Lobb et al. This content is distributed under the terms of the Creative Commons Attribution 4.0 International license.

Previous research showed that Acetobacteroides hydrogenigenes RL-C can produce acetate and carbon dioxide from glucose fermentation (28). Because these metabolites could potentially be converted to methane by methanogenic archaea (52), we analyzed the assembled metagenomic data for archaea-associated contigs. Contigs taxonomically affiliated with methanogenic archaea were identified from almost all samples, including species of the classes *Methanobacteria*, *Methanococci*, and *Methanomicrobia* (Fig. S7a). A 1.7-fold-higher relative abundance of these contigs was observed after day 1 (Fig. S7a), indicating that methanogenic archaea may have increased in relative abundance early in decomposition, coinciding with anoxic conditions and the generation of acetate or carbon dioxide by *Rikenellaceae* bacteria. However, no archaeal taxa were identified in the 16S rRNA. This potentially reflects an increased ability to detect low-abundance archaeal organisms in our metagenomic data set, perhaps due to the availability of more taxonomic markers and/or lower archaeal 16S copy numbers (53).

10.1128/mSystems.00145-20.7 FIG S7 Relative abundance of methanogen contigs and metagenomic bins. (a) Relative abundance (calculated by mean contig coverage/mean sample coverage) of archaeal contigs belonging to the methanogen classes *Methanobacteria*, *Methanococci*, and *Methanomicrobia*. (b) Relative abundance (calculated by mean bin coverage/mean sample coverage) of the four high-quality bins assembled from the fish necrobiome community as well as a small incomplete bin, Bin_11. Download FIG S7, TIF file, 0.8 MB. Copyright © 2020 Lobb et al. 2020 Lobb et al. This content is distributed under the terms of the Creative Commons Attribution 4.0 International license.

### A toxigenic strain of Aeromonas veronii is a dominant member of the necrobiome.

**FIG 7:**
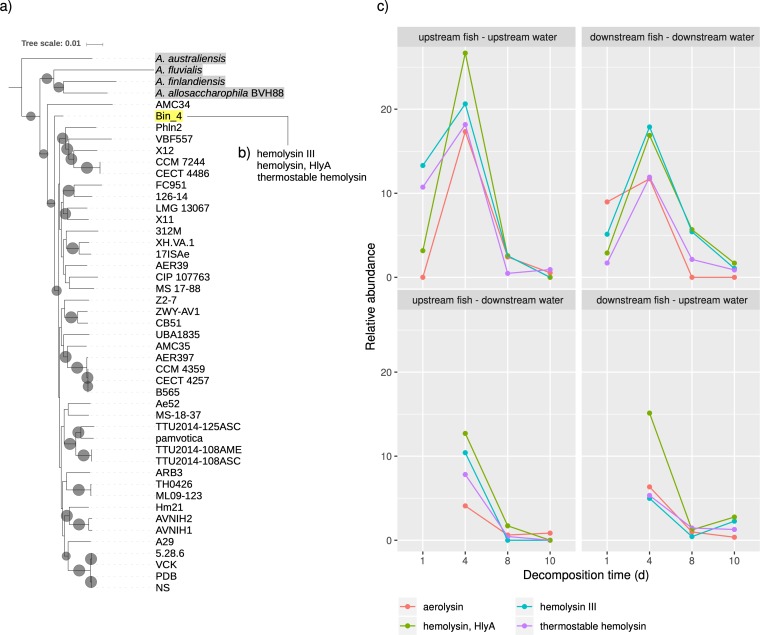
A toxigenic Aeromonas veronii-like strain is a dominant species in early decomposition. (a) RAxML tree using the GTR+GAMMA model made from concatenated single-copy core gene nucleotide sequences detected with Anvi’o (Campbell et al. set [70] ). The tree was outgrouped on Aeromonas hydrophila. Gray circles are scaled to bootstrap support of ≥85, with the largest size representing 100. *Aeromonas* species outside Aeromonas veronii are highlighted in gray. Representative Aeromonas veronii strains from the NCBI Genome Tree report were chosen to display here (not highlighted), and only their strain name is shown. This tree was visualized with iTOL (69). (b) Bin_4’s predicted toxin repertoire from VFDB. (c) Relative abundance (mean gene coverage/mean sample coverage) of *Aeromonas* hemolysin toxin genes. Decomposition time is shown in days.

Because Bin_4 affiliated with A. veronii, a well-established pathogen of fish and humans (36, 54–60), and a common inhabitant of the fish gut microbiome (40–43), we explored its phylogenetic position, functional profile, and virulence repertoire. A maximum likelihood phylogeny of A. veronii and other related *Aeromonas* genomes from the NCBI was constructed based on a concatenated alignment of conserved ribosomal marker genes (Fig. 7a). Within this phylogeny, Bin_4 grouped with a clade of *A. veronii* genomes but as a basal lineage outgrouping all *A. veronii* species except AMC34.

We used the VFanalyzer from the Virulence Factor Database (VFDB) to detect virulence factors within Bin_4 and compared it to a reference *Aeromonas* strain, *A. veronii* B565. This bin contained virulence-related genes for adherence, iron uptake, and secretion systems (Data Set S1C). Indeed, a total of 54 genes that were associated with secretion systems were identified, compared to only 15 in *A. veronii* B565. In addition, we identified 13 genes associated with endotoxin production. Like *A. veronii* B565, Bin_4 genes encoded hemolysin III, hemolysin HlyA, and a thermostable hemolysin gene (Fig. 7b). We also recovered a relatively small incomplete bin (Bin_11, 0.64 Mb, 717 coding sequences [CDSs], 321 contigs) that correlated with Bin_4 in relative abundance (Fig. S7b). This small bin affiliated with Aeromonas veronii and also included a gene encoding aerolysin toxin production (Data Set S1D). Based on metagenomic mapped read coverage, the relative abundance of genes encoding *Aeromonas* toxins increased on day 4 of decomposition (Fig. 7c), indicating an enrichment in *Aeromonas* strains carrying hemolytic proteins. A possible explanation for this is that lytic toxins, including those from *Aeromonas*, may function in host cell lysis during decomposition and therefore peak in relative abundance during earlier stages of decomposition. Bin_4 also possessed genomic potential for decomposition-related pathways, including histidine degradation (contains EC 4.3.1.3, 4.2.1.49, 3.5.2.7, and 3.5.3.8) and the production of putrescine (EC 4.1.1.19, 3.5.3.12, and 3.5.1.53), indole (EC 4.1.99.1), and cadaverine (EC 4.1.1.18).

### Conclusion.

Overall, our microcosm study of rainbow darter fish decomposition revealed a highly reproducible microbial succession throughout the time course, even across different fish and water/sediment sources. The location of the fish when sampled (upstream or downstream of the WWTP) also affected its decomposition profile, suggesting that necrobiomes may be influenced by prior fish-environment interactions. Together, our data suggest that environmental interactions may shape the initial gut community and/or the physiological state of the fish, which then seeds or impacts the later necrobiome community and its succession.

Both 16S and metagenomic analysis revealed a strong succession in which initial time points were dominated by *Clostridiaceae* and *Aeromonas*, with *Rikenellaceae* species appearing by day 4 and becoming major community members by day 10. Analysis of functional profiles inferred from the metagenomic data revealed common decomposition pathways, as well as temporal shifts in function that mirrored taxonomic succession. Notably, pollutant degradation pathways and biofilm formation pathways were enriched in the early stages of decomposition and associated with *Clostridiaceae* and *Aeromonas*, and glycan metabolism and antibiotic synthesis increased in later stages and associated with *Rikenellaceae*. Last, we identified a toxigenic *Aeromonas* strain that was a dominant member of the necrobiome community. The presence of numerous hemolytic toxin genes in this organism suggests a potential role for toxins in the decomposition of host tissues as proposed previously (11). Further work investigating the prevalence and function of toxigenic bacterial species in decomposer communities will be important to explore their broader ecological roles and niches within natural ecosystems.

## MATERIALS AND METHODS

### Fish collection.

On 24 October 2016, female rainbow darters (*Etheostoma caeruleum*) were collected from the Grand River (Fig. 1), both upstream (Westmontrose [WMR]; 43°35′08′′N; 80°28′53′′W) and downstream (Economic Insurance Trail [EIT]; 43°28′24′′N; 80°28′22′′W) of the Waterloo wastewater treatment plant (WWTP) (43°29′16′′N; 80°30′25′′W). Forty-two fish (21 from each site) were collected using a backpack electrofisher (Smith Root, LR-20) and euthanized quickly with a sharp blow to the head. Then each fish was placed in an autoclaved 250-ml mason jar microcosm that contained a mixture of water and river substrate (see Table S1 for river water quality metadata and Fig. S1a for an example mason jar setup). The lids were closed, but not sealed, in order to ensure oxic conditions that would accompany natural in-river decay events. The jars were then left to decay in a fume hood at room temperature. Three samples containing both fish and water/sediment from the same site were left to decompose for 1 day (24 h), 4 days, 8 days, and 10 days for both the WMR and EIT sites, totaling 24 fish. For additional treatments to assess differences in water quality and aquatic microorganisms, three samples containing fish and water/sediment from different sites (i.e., WMR fish in EIT conditions and EIT fish in WMR conditions) were allowed to decay for 4, 8, and 10 days, totaling 18 fish. At each time point, decay was documented (Fig. S1b), and fish were removed from the replicate jars, then rinsed with sterile water, and ground with liquid nitrogen using a clean mortar and pestle. The powdered tissue was stored at –80°C prior to genomic DNA extraction.

10.1128/mSystems.00145-20.8 TABLE S1 Water quality parameters from collection sites taken on 24 October 2016 upstream (43°35′08′′N; 80°28′53′′W) and downstream (43°28′24′′N; 80°28′22′′W) from the Waterloo wastewater treatment plant (WWTP; 43°29′16′′N; 80°30′25′′W). Data are presented as the means plus 1 standard deviation (STD) from 24 samples (hourly) from remote monitoring stations located along the Grand River. Data were obtained through the Grand River Conservation Authority (www.grandriver.ca). Download Table S1, PDF file, 0.05 MB. Copyright © 2020 Lobb et al. 2020 Lobb et al. This content is distributed under the terms of the Creative Commons Attribution 4.0 International license.

10.1128/mSystems.00145-20.9 TABLE S2 Differentially abundant ASVs between downstream WWTP and upstream WWTP necrobiome samples. Download Table S2, PDF file, 0.08 MB. Copyright © 2020 Lobb et al. 2020 Lobb et al. This content is distributed under the terms of the Creative Commons Attribution 4.0 International license.

Experimental procedures and the use of animals in this study were approved by the University of Waterloo Animal Care Committee and within Canadian Council on Animal Care (CCAC) guidelines (AUPP 40318).

### DNA extraction.

Unless noted, all chemicals and reagents were purchased from Sigma-Aldrich (Mississauga, Ontario, Canada). For DNA extraction, 100 mg of ground tissue was added to 1.2 ml of TE buffer (10 mM Tris-HCl, 1 mM EDTA [pH 8.0]), 100 μl of 10% sodium dodecyl sulfate (SDS), 20 μl of proteinase K, 8 μl of RNase A, and 200 μl of 5 M NaCl. This mixture was vortexed quickly and incubated at 55°C for 30 min. Then 160 μl of CTAB extraction solution (2% cetrimonium bromide, 100 mM Tris, 20 mM EDTA, 1.4 M NaCl [pH 8.0]) was added, and the samples were further incubated at 65°C for 1.5 h. Following this lysis incubation, 700 μl of the lysate was extracted with an equal volume of phenol and centrifuged at 10,000 × *g* for 5 min. The aqueous phase was retained and twice extracted with equal volumes of phenol-chloroform-isoamyl alcohol (25:24:1), followed each time with centrifugation at 10,000 × *g* for 5 min. One volume of isopropanol was used to precipitate aqueous phase DNA in a new ultracentrifuge tube, followed by centrifugation at 13,000 × *g* for 10 min at room temperature. The resulting pellet was washed twice with 70% ethanol, dried, and then dissolved in 50 μl of DNase- and RNase-free H_2_O (Sigma) at 50°C for 15 min. The quantity and quality of DNA were determined with a SpectraDrop (Molecular Devices) and stored at –20°C prior to sequencing.

### 16S rRNA gene and metagenomic sequencing.

Extracted DNA was amplified in triplicate using Pro341F and Pro805R universal prokaryotic primers (61). Triplicate amplicons were pooled, gel quantified, and sequenced to a depth of at least 30,000 paired-end reads per sample using the MiSeq reagent kit v3 (2 × 300 cycles; Illumina).

For metagenomic sequencing, genomic DNA (1 ng) was fragmented and individually barcoded using the Nextera XT DNA Library Prep kit (Illumina) following the supplier’s guidelines. Small fragments of library DNA were removed by adding 0.6 volumes of AMPure XP beads (Beckman Coulter). After washing twice with 80% ethanol and air drying for 10 min, DNA was eluted from the beads with 10 mM Tris-HCl (pH 8.5). Purified library DNA was quantified with the Qubit dsDNA (double-stranded DNA) HS (high-sensitivity) assay kit, diluted to 4 nM with the Tris-HCl buffer and then pooled in an equal volume. Library DNA was denatured with equal volumes of 0.2 N NaOH, diluted to 7 pM with hybridization buffer HT1, and sequenced with MiSeq reagent kit v2 (2 × 250 cycles; Illumina).

### 16S rRNA gene analysis.

Demultiplexed sequences were processed using DADA2 v1.4 (62), managed through QIIME2 v.2017.10 (63). Briefly, forward and reverse reads were truncated with decreasing quality metrics while maintaining sequence overlap (∼250 bases). Primers were removed, and paired reads were assembled after error modeling and correction, creating amplicon sequence variants (ASVs). Chimeric ASVs were removed by reconstruction against more abundant parent ASVs. The resulting ASV table was constructed for downstream analysis (see Data Set S1A in the supplemental material).

Taxonomy was assigned to representative sequence variants using a naive Bayesian classifier implemented in QIIME2 with scikit-learn (v.0.19.0), trained against SILVA release 128 (64), clustered at 99% identity, and trimmed to the amplified region. Assignments were accepted above a 0.7 confidence threshold.

For ordination, we used a proportion matrix of ASVs across each sample with a sparsity cutoff (i.e., ASV detected in at least 3 of 42 samples). The metaMDS() and envfit() scripts from vegan package v2.4-2 in R were used to calculate ordination coordinates and data vectors. A stress or Shepard diagram was generated with stressplot() from the vegan package to determine the nonmetric fit. The ASVs with significant rank sum differences in sample proportion were calculated with the Mann-Whitney test in R. Multiple hypothesis correction of *P* values was performed using the p.adjust() function in R with the Benjamini-Hochberg model. We also calculated differential taxon relative abundance using a variety of methods (metagenomeSeq, edgeR, DESeq2, and LEfSe) as implemented in the Marker Data Profiling pipeline from MicrobiomeAnalyst (65) with default settings on 20 March 2020.

### Metagenomic data analysis.

Raw reads were processed with TrimGalore v0.5.0, coassembled with metaSPAdes (SPAdes v3.12.0), and eukaryotic contigs were identified with Centrifuge v1.0.4 using their NCBI nr preindexed database (last updated 3 March 2018) and subsequently removed. Reads were mapped with Bowtie 2 v2.3.4.3 using default settings and binned using CONCOCT with Anvi’o v5.2 (minimum 1-kb contig cutoff). Mean coverage data for the metagenomic functional analyses and for the methanogen analysis were extracted from Anvi’o (66) using all contigs (no contig length cutoff).

For metagenomic and bin functional analysis, KEGG (Kyoto Encyclopedia of Genes and Genomes) annotations were identified with GhostKOALA (67). The average coverage for each gene (per base pair), normalized by dividing by the average sample coverage (per base pair), was summed to give a total coverage value for each KEGG pathway. The decostand() function from the vegan package v2.4-2 in R was used to determine the fractional value of each pathway with respect to the total summed coverage across all KEGG pathways detected in the sample. A Kruskall-Wallis test was done in R to identify KEGG pathways with significantly different distributions by day of decomposition. The decostand() function was also used to proportionally normalize each pathway value across every sample for plotting. For the bin functional analysis, the frequency of each KEGG orthology (KO) annotation in each MAG bin was counted. These counts were summed for each KEGG pathway, and fractional values were calculated across all KEGG pathways detected in the bins as before.

The VFanalyzer software from the Virulence Factor Database (VFDB) (68) identified virulence factors in the predicted coding sequences of Bin_4 using Aeromonas veronii B565 as a representative genome. We also used the domain architecture from the *Aeromonas* toxin gene set from the VFDB to identify *Aeromonas* toxin genes in the coassembly. Putative toxins longer than 150 amino acids were assessed with BLASTp for *Aeromonas* taxonomy and gene annotation.

### Data availability.

All 16S rRNA gene and metagenomic sequencing data for this project were deposited into the NCBI Short Read Archive (SRA) under BioProject accession no. PRJNA604775.
