# Supplementary material for: Time Series Resolution of the Fish Necrobiome Reveals a Decomposer Succession Involving Toxigenic Bacterial Pathogens
Source: mSystems. 2020 Apr 28;5(2):e00145-20. doi: 10.1128/mSystems.00145-20 (PMC7190384; doi:10.1128/mSystems.00145-20)
Supplement: TABLE S1 [file mSystems.00145-20-st001.pdf]

	**Temperature (^oC)**	**Dissolved Oxygen (mg/L)**	**pH**	**Conductivity (µS/cm)**	**Nitrate (mg/L)**
Upstream	14.3 ± 0.2	12.4 ± 1.2	8.5 ± 0.1	339.8 ± 2.7	Not Measured
Downstream	10.5 ± 0.4	10.5 ± 0.9	8.8 ± 0.1	502.2 ± 3.7	1.19 ± 0.01
