# Supplementary material for: Time Series Resolution of the Fish Necrobiome Reveals a Decomposer Succession Involving Toxigenic Bacterial Pathogens
Source: mSystems. 2020 Apr 28;5(2):e00145-20. doi: 10.1128/mSystems.00145-20 (PMC7190384; doi:10.1128/mSystems.00145-20)
Supplement: TABLE S2 [file mSystems.00145-20-st002.pdf]

**ASVs more abundant downstream vs. upstream WWTP samples**	**Mann-Whitney adjusted *p*-value (one-tailed)**	**Taxonomy (if ASV is not clear)**
Arcobacter_62	$1.22x10^{-4}$	
Acidaminobacter_71	$1.60\ x10^{-4}$	
Anaerosinus_159	$3.08\ x10^{-3}$	
Peptostreptococcaceae_74	$5.82\ x10^{-3}$	
Pseudomonas_77	$6.73\ x10^{-3}$	
Acetobacteroides_16	$1.06\ x10^{-2}$	
Acetobacteroides_27	$1.06\ x10^{-2}$	
Shewanella_158	$1.06\ x10^{-2}$	
Ambiguous_taxa_266	$1.06\ x10^{-2}$	D_0__Bacteria; D_1__Proteobacteria; D_2__Epsilonproteobacteria; D_3__Campylobacterales; D_4__Campylobacteraceae; D_5__Sulfurospirillum; Ambiguous_taxa
JTB215_41	$1.28\ x10^{-2}$	D_0__Bacteria; D_1__Firmicutes; D_2__Clostridia; D_3__Clostridiales; D_4__JTB215
Hydrogenoanaerobacterium_218	$2.05\ x10^{-2}$	
Acetobacteroides_1	$2.25\ x10^{-2}$	
Proteocatella_25	$3.50\ x10^{-2}$	
Sedimentibacter_202	$3.50\ x10^{-2}$	
Acidovorax_225	$3.50\ x10^{-2}$	
Ambiguous_taxa_365	$7.28\ x10^{-2}$	D_0__Bacteria; D_1__Proteobacteria; D_2__Betaproteobacteria; D_3__Burkholderiales; D_4__Comamonadaceae; D_5__Acidovorax; Ambiguous_taxa
bacterium enrichment culture clone M624_205	$9.61\ x10^{-2}$	D_0__Bacteria; D_1__Chlorobi; D_2__Chlorobia; D_3__Chlorobiales; D_4__OPB56; D_5__bacterium enrichment culture clone M624;

		D_6__bacterium enrichment culture clone M624
Peptostreptococcaceae_39	1.03 x10^{-1}	
Macellibacteroides_129	1.03 x10^{-1}	
Macellibacteroides_214	1.03 x10^{-1}	
Ambiguous_taxa_176	1.09 x10^{-1}	D_0__Bacteria; D_1__Proteobacteria; D_2__Gammaproteobacteria; D_3__Aeromonadales; D_4__Aeromonadaceae; D_5__Tolumonas; Ambiguous_taxa
**ASVs more abundant upstream vs. downstream WWTP samples**	**Mann-Whitney adjusted *p*-value (one-tailed)**	
Acidaminobacter_82	1.21 x10^{-3}	
Acetobacteroides_6	3.84 x10^{-2}	
Pelosinus_130	3.84 x10^{-2}	
Fonticella_58	4.10 x10^{-2}	
Pelosinus_68	4.10 x10^{-2}	
Shewanella_208	4.10 x10^{-2}	
